# Identification of Flavonoids as Putative ROS-1 Kinase Inhibitors Using Pharmacophore Modeling for NSCLC Therapeutics

**DOI:** 10.3390/molecules26082114

**Published:** 2021-04-07

**Authors:** Shraddha Parate, Vikas Kumar, Jong Chan Hong, Keun Woo Lee

**Affiliations:** 1 Division of Applied Life Science, Plant Molecular Biology and Biotechnology Research Center (PMBBRC), Gyeongsang National University (GNU), 501 Jinju-daero, Jinju 52828, Korea; parateshraddha@gmail.com; 2 Division of Life Sciences, Department of Bio & Medical Big Data (BK21 Four Program), Research Institute of Natural Science (RINS), Gyeongsang National University (GNU), 501 Jinju-daero, Jinju 52828, Korea; vikaspathania777@gmail.com

**Keywords:** ROS-1 kinase, drug resistance, NSCLC, flavonoids, structure-based pharmacophore, virtual screening, molecular docking, molecular dynamics simulations, MM/PBSA

## Abstract

Non-small cell lung cancer (NSCLC) is a lethal non-immunogenic malignancy and proto-oncogene ROS-1 tyrosine kinase is one of its clinically relevant oncogenic markers. The ROS-1 inhibitor, crizotinib, demonstrated resistance due to the Gly2032Arg mutation. To curtail this resistance, researchers developed lorlatinib against the mutated kinase. In the present study, a receptor-ligand pharmacophore model exploiting the key features of lorlatinib binding with ROS-1 was exploited to identify inhibitors against the wild-type (WT) and the mutant (MT) kinase domain. The developed model was utilized to virtually screen the TimTec flavonoids database and the retrieved drug-like hits were subjected for docking with the WT and MT ROS-1 kinase. A total of 10 flavonoids displayed higher docking scores than lorlatinib. Subsequent molecular dynamics simulations of the acquired flavonoids with WT and MT ROS-1 revealed no steric clashes with the Arg2032 (MT ROS-1). The binding free energy calculations computed via molecular mechanics/Poisson-Boltzmann surface area (MM/PBSA) demonstrated one flavonoid (Hit) with better energy than lorlatinib in binding with WT and MT ROS-1. The Hit compound was observed to bind in the ROS-1 selectivity pocket comprised of residues from the β-3 sheet and DFG-motif. The identified Hit from this investigation could act as a potent WT and MT ROS-1 inhibitor.

## 1. Introduction

Lung cancer remains one of the fatal causes of cancer related malignancies in the world with non-small cell lung cancer (NSCLC) accounting for 85% of cases [1]. NSCLC encompasses numerous subtypes, including lung adenocarcinoma (LUAD) and lung squamous cell carcinoma (LUSC) and despite advances in diagnosis, 70% of patients present with metastatic lung cancer disease when surgery is not an option [2]. Receptor tyrosine kinases (RTKs) have emerged as critical players of cellular communication function and network. Activating fusions and rearrangement of the proto-oncogene ROS-1 (c-ros oncogene 1) have been reported in 1–2% of NSCLC cases as potent oncogenic drivers, often found in non-smokers and patients of younger age [3]. Furthermore, oncogenic ROS-1 leads to the activation of several components of the PI3K/AKT/mTOR signaling pathway [4]. Numerous ROS-1 fusion proteins have been identified and reported in literature including SDC4, TMEM106B-ROS-1, SLC34A2-ROS-1, TPM3, CD74-ROS-1, LRIG3, EZR, CLTC, TPD52L1-ROS-1, CCDC6 and FIG-ROS-1 [5,6,7,8].

Many researchers are pouring tremendous efforts into developing effective and efficient ROS-1 inhibitors [9]. The ROS-1 gene has 49% sequence homology in the kinase domain and 77% identity at the ATP-binding site with anaplastic lymphoma kinase (ALK), thereby forming the basis for the utilization of ALK inhibitors for ROS-1 rearranged NSCLC patients [10]. To date, most of the ROS-1 inhibitors with remarkable in vitro inhibitory activities are the repurposing of first- and second-generation inhibitors of ALK, including alectinib, ceritinib, foretinib and crizotinib [11,12,13,14]. The first-generation ALK inhibitor, crizotinib (Xalkori^®^, Pfizer), was granted FDA approval in 2016 for the treatment of ROS-1 rearranged NSCLC [9]. However, the insurgence of Gly2032Arg mutation in the ROS-1 kinase domain conferred resistance to crizotinib treatment in ROS-1 fusion-positive NSCLC patients [15]. The Gly2032 residue at the solvent front in the rigid phosphate-binding loop (P-loop) of the kinase domain interacts with the pyrazole group of crizotinib via van der Waals forces. The mutated Arg2032 residue partially occupies the pyrazole binding space, thereby interfering with crizotinib binding and leading to resistance and steric clashes with its piperidine ring [16]. To overcome this, a third-generation ROS-1/ALK inhibitor, lorlatinib (PF 06463922, Pfizer), was designed lacking the piperidine ring, which was replaced by a pyridine ring for preventing the Arg2032-induced steric clashes [16]. Additional structural changes to handle drug resistance were also made in lorlatinib leading to better therapeutic efficacy than crizotinib. Another tyrosine kinase inhibitor, Entrectinib (ROZLYTREK, Genentech Inc.), was approved by FDA in 2019 for ROS-1 altered lung cancers [17]. The multi-kinase inhibitor, Cabozantinib, also demonstrated effective clinical efficacy in ROS-1 positive NSCLC patients [18]. Despite demonstrating efficient activity against ROS-1 cancers, the aforementioned drugs showed limited efficacy against Gly2032Arg ROS-1 mutants [19]. Therefore, there is an emergent need to develop efficient new inhibitors, selective for ROS-1 that would overcome Gly2032Arg-mediated resistance.

Phytochemicals are plant-derived natural compounds used to treat several ailments, including cancer, and numerous reports have demonstrated their influence on tumor proliferation and metastasis via in vitro and in vivo studies [20]. Moreover, natural products exhibit greater potential druggable pharmacophores when compared to synthetic compounds characterized by their wide availability, cost-effectiveness and high tolerance [20]. These polyphenols comprise flavonoids, stilbenes, lignans and phenolic acids among which flavonoids constitute approximately two-thirds of plant polyphenols in the human diet [21]. Natural flavonoids categories, including flavonols, anthocyanins, isoflavones and flavones, exert beneficial effects on human health and dietary intake and represent a novel therapeutic template for the design of cancer inhibitors [20]. Furthermore, flavonoids scavenge the free radicals involved in DNA damage and tumor progression [22]. The protective ability of some of the flavonoids is well-documented in the literature with quercetin and naringin as examples [22]. A study published recently reported on Trifolium flavonoids in overcoming gefitinib resistance of NSCLC by downregulating the STAT3 and ERK pathways [23]. The anti-cancer effects of flavonoids on lung cancer cell lines were also reported via inhibition of the Wnt/β-catenin signaling pathway [24]. Flavonoids have also exhibited cytotoxic potential against lung cancer cell lines in several studies by arresting the progression of the cell cycle and inducing cancer cell apoptosis [25,26,27]. More importantly, the structural backbone of flavonoids contributes to the killing of resistant cancer cells [28].

On the basis of the above-mentioned perspectives, we sought to identify natural product flavonoids as potential therapeutics targeted to ROS-1 tyrosine kinase for the NSCLC treatment. We have carried out an in silico study considering the development of a receptor-based pharmacophore model for virtual screening by adopting the ROS-1-lorlatinib interaction as a template structure. The developed pharmacophore model has been utilized to virtually screen flavonoids from the TimTec database, and subsequently, the acquired flavonoids were filtered for their drug-likeness via Lipinski’s, Veber’s and ADMET filters. The binding interaction of obtained drug-like flavonoids was investigated with the ROS-1 kinase domain pocket residues. Additionally, the flavonoids obtained from docking analysis with ROS-1 kinase domain were subjected to extensive molecular dynamics (MD) simulations to check their stability over a period of 50 ns. The flavonoids demonstrating binding interactions with key residues of the ROS-1 kinase domain similar to that observed in the ROS-1-lorlatinib crystallographic structure were additionally docked with the constructed Gly2032Arg ROS-1 mutant structure. The flavonoids are exhibiting interactions with Gly2032 and Arg2032 in wild type (WT) and mutant (MT) ROS-1, respectively, which were identified as molecules with potent scaffolds that could inhibit ROS-1. Furthermore, the binding free energy (BFE) calculations executed through MM/PBSA (molecular mechanics/Poisson-Boltzmann surface area) displayed effective binding affinity of flavonoids with WT and MT ROS-1 kinase domain.

## 2. Results

### 2.1. Receptor-Ligand Pharmacophore Model

**Figure 1:**
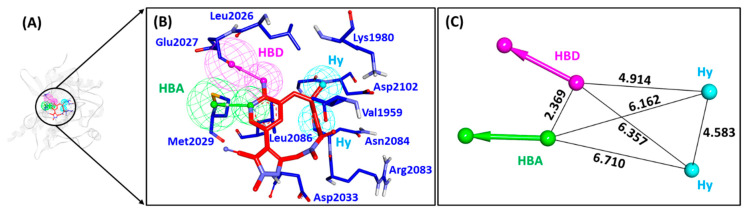
Receptor-ligand pharmacophore model- Pharmacophore_01. (**A**) Pharmacophore model generated at the catalytic site of c-ros oncogene1 (ROS-1) complexed with its co-crystallized ligand, lorlatinib. (**B**) ROS-1 selective inhibitor, lorlatinib mapping with essential residues of the ROS-1 active site via key pharmacophoric features- HBD, HBA and Hy. (**C**) Interfeature distance between the generated features of the pharmacophore. HBD (hydrogen bond donor); HBA (hydrogen bond acceptor); Hy (hydrophobic).

A receptor-ligand (structure-based) pharmacophore model was generated from the crystallographic structure of ROS-1 tyrosine kinase complexed with its selective inhibitor, lorlatinib (PDB ID: 4UXL) [16]. The ROS-1 selective inhibitor, lorlatinib exhibited improved activity (IC_50_ = 0.6 nM) against ROS-1 compared to dual ALK/ROS-1 inhibitor, crizotinib (IC_50_ = 253.7 nM) and also demonstrated in vivo activity in patients expressing Gly2032Arg mutation [16]. The key pharmacophoric features of lorlatinib binding with ROS-1 were explored, resulting in the generation of only one pharmacophore hypothesis with one hydrogen bond donor (HBD), one hydrogen bond acceptor (HBA), and two hydrophobic (Hy) features as essential features (Table 1, Figure 1). The Pharmacophore_01 with a selectivity score of 7.0747 indicated that lorlatinib interacts with Glu2027 and Met2029 via HBD and HBA features, respectively forming hydrogen bond interactions, while hydrophobic interactions with residues Val1959 (alkyl), Ala1978 (π-alkyl), Lys1980 (alkyl), Leu2026 (alkyl) and Leu2086 (π-sigma) are observed via Hy pharmacophoric feature. Lorlatinib additionally formed several van der Waals interactions with residues including Leu2010, Gly2032, Asp2033, Arg2083 and Asp2102. A study by Pathak et al. reported the structure-based pharmacophore model where they screened two commercial databases- Maybridge and Chembridge. The authors were able to successfully acquire five hits with the aforementioned pharmacophoric features, capable of inhibiting the WT and MT ROS-1 proteins [29]. Therefore, the Pharmacophore_01 model was taken forward for screening the database of flavonoids.

### 2.2. Güner-Henry Validation of the Receptor-Ligand Pharmacophore Model

The Pharmacophore_01 model generated above was validated via Güner-Henry method, also referred to as decoy set validation, used for measuring the overall suitability of the model in picking active compounds from decoys. An external database (D) of 78 molecules with 20 actives (A) ROS-1 inhibitors was prepared with the remaining molecules as decoy molecules. The Pharmacophore_01 model was able to retrieve 25 hits (Ht) with 20 active compounds (Ha), resulting in the goodness of fit (GF) score of 0.77. The GF score was observed to be near the ideal model range value [30], thereby confirming the selectivity of our model in picking active compounds from decoys (Table 2).

### 2.3. Drug-Like Flavonoids Retrieved by Virtual Screening

**Figure 2:**
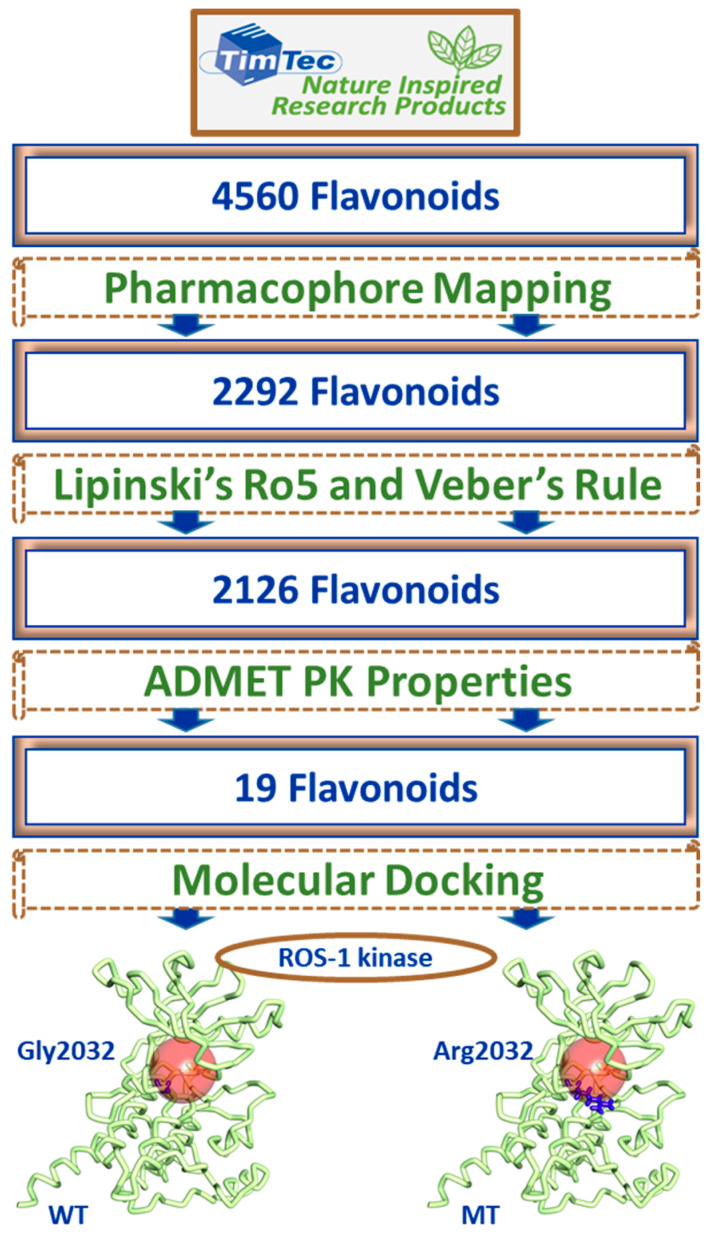
Representation of the steps involved in retrieving drug-like flavonoids for molecular docking, from the TimTec database using the receptor-ligand pharmacophore model.

The well-validated Pharmacophore_01 model was used as a 3D query to screen the TimTec database of 4560 flavonoids, mapping a total of 2292 compounds. Subsequently, three filters were applied to the obtained compounds to achieve drug-like flavonoids. A total of 2126 flavonoids passed the Lipinski’s rule of five (Ro5) and Veber’s filter and this number was further reduced to 19 flavonoids following the absorption, distribution, metabolism, excretion and toxicity (ADMET) pharmacokinetic (PK) properties. These 19 flavonoid compounds were taken forward for the process of molecular docking with ROS-1 kinase domain along with co-crystallized ligand, lorlatinib as a reference compound (Figure 2). The docking scores and interactions of flavonoids with the residues of the ROS-1 kinase domain catalytic pocket (Leu1951, Gly1952, Gly1954, Val1959, Glu1961, Lys1976, Ala1978, Lys1980, Leu2010, Leu2026, Glu2027, Leu2028, Met2029, Glu2030, Gly2032, Asp2033, Glu2061, Arg2083, Asn2084, Leu2086, Gly2101 and Asp2102) were analyzed and compared with that of lorlatinib.

### 2.4. Molecular Docking of Retrieved Flavonoids with ROS-1 Tyrosine Kinase Domain

The drug-like flavonoids derived from the virtual screening of the TimTec database were subjected to molecular docking analysis with the catalytic site of the ROS-1 tyrosine kinase domain. Before docking of flavonoids, the capability of GOLD docking software was assessed by re-docking of the co-crystallized ligand, lorlatinib in the ROS-1 binding pocket. This process resulted in an acceptable root-mean square deviation (RMSD) of 0.83 Å (Figure S1) between the docked pose and available lorlatinib co-crystallized pose. Consequently, the docking of 19 flavonoid molecules was carried out to evaluate their interactions with the aforementioned residues of the ROS-1 catalytic site. With a Gold score of 43.74 for lorlatinib, a total of 10 flavonoids displayed docking scores higher than the reference compound and were observed to interact with the key residues of the ROS-1 kinase domain (Table S1). More particularly, the interactions of acquired flavonoids with Gly2032 in the WT ROS-1 kinase domain were observed, as characterized by van der Waals interaction. These 10 flavonoids, along with lorlatinib, were also docked with the MT ROS-1 kinase domain, where Gly2032 was mutated to Arg2032. A total of nine flavonoids demonstrated higher docking scores and favorable interactions with the mutated kinase domain (Table S2). In particular, the nine compounds formed interactions with Arg2032 via hydrogen or hydrophobic bonds. Furthermore, the binding stability of the identified flavonoids with WT and MT ROS-1 kinase domain was assessed for steric clashes via MD simulations in the next step.

### 2.5. Binding Mode, Interaction and Free Energy Analysis of Identified Flavonoids by Molecular Dynamics Simulations

**Figure 3:**
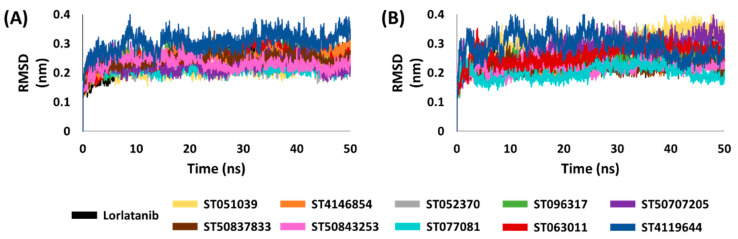
Backbone root-mean-square deviation (RMSD) analysis of (**A**) wild-type (WT) and (**B**) mutated (RT) ROS-1 systems.

MD simulations were employed for the identified flavonoids and lorlatinib with the WT and MT ROS-1 kinase to check their stabilities for 50 ns. A total of 22 docking systems (11 WT + 11 MT) were taken as initial co-ordinates for MD. The binding stability of the flavonoids was analyzed in terms of their backbone RMSD plots. The RMSD values were observed to be in the range of 0.1–0.4 nm, indicating the stability of the flavonoids with both WT and MT kinase domains (Figure 3).

**Figure 4:**
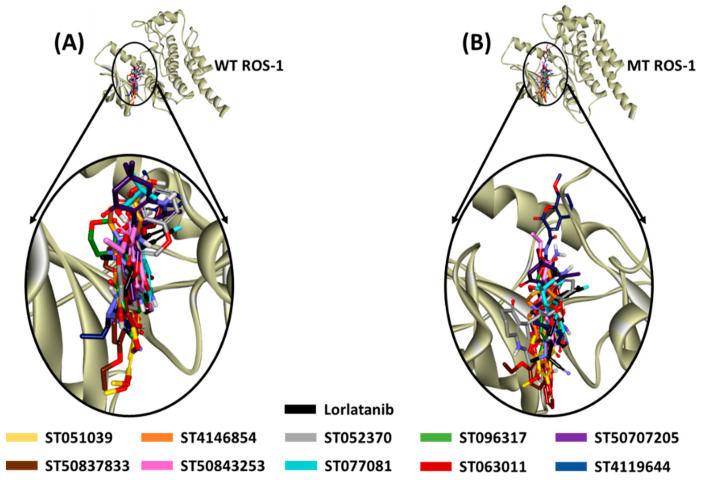
Binding mode of co-crystallized inhibitor, Lorlatinib (reference) and identified flavonoids within the catalytic pocket of (**A**) WT and (**B**) MT ROS-1 kinase.

The representative structures from the last 10 ns of stable MD trajectories were extracted and superimposed to observe the binding mode of the flavonoids in both the WT and MT ROS-1 kinase catalytic pocket. The flavonoids were perceived to exhibit a similar binding mode as the ROS-1 co-crystallized inhibitor, lorlatinib (Figure 4). The binding interaction of the acquired flavonoids with both the WT and MT ROS-1 further indicated that the compounds formed requisite bonds with the aforementioned residues of the binding pocket as characterized by hydrogen, van der Waals and hydrophobic interactions (Figures S2 and S3). Furthermore, the compounds did not show steric clashes with the Arg2032 residue of the MT ROS-1 kinase domain.

Additionally, the BFE score of the 11 flavonoids with WT and MT ROS-1 was calculated via MM/PBSA and compared with the BFE score of lorlatinib. With the BFE score of −88.244 ± 12.933 kJ/mol for lorlatinib binding with WT ROS-1, a total of three flavonoids demonstrated better BFE scores (Table S3, Figure S4A). In addition, the BFE score of lorlatinib with MT ROS-1 was computed as −75.505 ± 12.053 kJ/mol, and two flavonoids exhibited superior BFE scores (Table S4, Figure S4B). Only one flavonoid (hereafter referred to as Hit molecule) presented with better BFE scores both with WT (−91.685 ± 20.795 kJ/mol) and MT (−103.035 ± 16.223 kJ/mol) ROS-1 kinase than the aforesaid lorlatinib’s BFE with the kinase domains.

**Figure 5:**
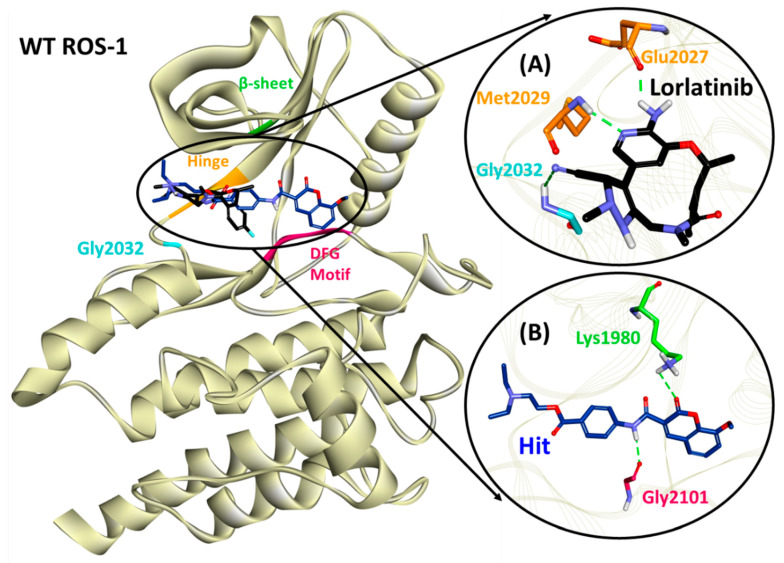
Binding mode of (**A**) lorlatinib and (**B**) Hit compound in the wild type (WT) ROS-1 catalytic pocket and molecular interactions with key residues.

**Figure 6:**
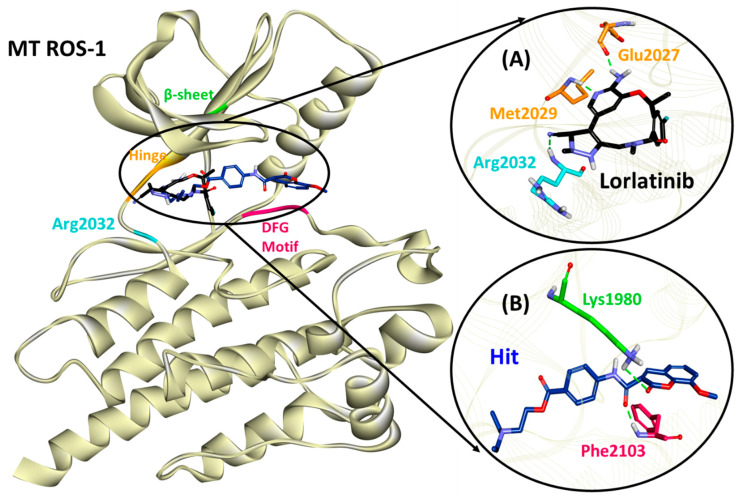
Binding mode of (**A**) lorlatinib and (**B**) Hit compound in the mutated (MT) ROS-1 catalytic pocket and molecular interactions with key residues.

The hydrogen bonds from the equilibrium trajectory of the lorlatinib’s interaction with both WT and MT ROS-1 systems were analyzed. Lorlatinib displayed key hydrogen bonding interactions with hinge residues- Glu2027 and Met2029 and also with the solvent front residue Gly2032 (Figure 5, Figure S2 and Table 3). Moreover, no steric clashes with Arg2032 (MT ROS-1) were observed (Figure 6 and Figure S3) as already reported in the literature, thereby explaining the effectivity of lorlatinib in inhibiting crizotinib-resistant NSCLC tumors. Upon scrupulous analysis, it was observed that the Hit molecule with the highest dock score of 72.80 and 73.62 with WT and MT ROS-1 (Tables S1 and S2), respectively maintained its hydrogen bonds with Lys1980 (from the β3 sheet) and Met2029 (from the hinge region) as seen in the docking analysis. However, the hydrogen bond with hinge residue Met2029 was weakened during 50 ns and gradually disappeared because of another hydrogen bond with Gly2101 (Hit/WT-ROS-1 interaction) (Figure 5, Figure S2 and Table 3) and Phe2103 (Hit/MT ROS-1 interaction) (Figure 6, Figure S3 and Table 3) from the DFG-motif. From the above meticulous analysis, it was observed that the Hit molecule inserts into DFG-motif, conferring a level of selectivity for ROS-1 over ALK as seen in previous studies by Tian et al. [31,32].

As a final assessment, the identified flavonoids were checked whether they were evaluated earlier for ROS-1 inhibitory activity, using the PubChem chemistry database (https://pubchem.ncbi.nlm.nih.gov/, accessed on 5 April 2021). The search results confirmed that the identified flavonoids, including our Hit compound were not tested experimentally for ROS-1 inhibition. The flavonoids also displayed key pharmacophoric features similar to lorlatinib, required for ROS-1 inhibition, as seen from the mapping analysis (Figure 1 and Figure S5). Altogether, these results determine that the acquired flavonoids, especially the Hit compound from the present study, can be recommended as potential inhibitors of ROS-1 kinase.

## 3. Discussion

The orphan RTK c-ros oncogene1 (ROS-1) is a molecular driver in NSCLC patients where approximately 15,000 new patients are estimated to harbor tumors driven by rearranged ROS-1 [14]. Crizotinib was observed to be highly effective against ROS-1 rearranged NSCLC tumors. However, the phylogenic proximity of ROS-1 and ALK led to crizotinib resistance due to the acquisition of Gly2032Arg mutation in the ROS-1 catalytic kinase domain [33]. Lorlatinib was therefore developed for preventing the Arg2032-mediated steric clashes, demonstrating effective activity against both WT ROS-1 (IC_50_ = 0.6 nM) and MT ROS-1 (IC_50_ = 203 nM) with marked selectivity for ROS-1 versus ALK [34,35].

The bioactive polyphenols exhibit therapeutic effects in cancers [36]. These polyphenols constitute flavonoids and non-flavonoids, among which the latter possesses antioxidant, anti-inflammatory and antibacterial activities. In carcinogenesis, flavonoids hinder multiple transduction pathways, limit the proliferation of cancer cells and increase apoptosis [37]. Moreover, they elevate the reactive oxygen species levels [37]. The therapeutic effectivity of flavonoids for the treatment of resistant EGF receptor-mutated NSCLC is discussed in detail [38]. The inhibitory activity of *Scutellaria* flavonoids was also observed in vitro and in vivo against the overexpression of Id1 in NSCLC [39]. The flavonoid cisplatin also potentiated anti-cancer activity in NSCLC A549 cells in vitro by inhibiting histone deacetylase [40]. The dietary flavonoid, kaempferol was also identified as a potent Nuclear factor erythroid 2-related factor 2 (Nrf2) inhibitor in NSCLC cells using Nrf2 reporter assay [41].

Encouraged from the above-mentioned efforts, we designed a pharmacophore-based virtual screening strategy [30,35,42,43,44,45,46] combined with molecular docking and molecular dynamics simulations to acquire flavonoids as effective WT and MT ROS-1 RTK inhibitors. Accordingly, a receptor-ligand pharmacophore model was developed deriving HBA, HBD and Hy as key pharmacophoric features (Figure 1). The generated pharmacophore model showed a good selectivity score of 7.0747 and was subsequently validated by the decoy set validation method, computing a GF score of 0.77. The GF score was acquired near the upper limit value of 1, thereby demonstrating the overall suitability of our model in evaluating active molecules from a given dataset [30,46]. A total of 4560 flavonoids from the *TimTec* database (https://www.timtec.net/, accessed on 5 April 2021) were screened with our model as query structure, obtaining 2292 flavonoids. Consequently, 19 drug-like flavonoids were retrieved after subjecting the 2292 flavonoids for their drug-likeness properties using Lipinski’s Ro5 and ADMET filters (Figure 2). Molecular docking of 19 flavonoids with the WT and MT ROS-1 RTK domain (PDB ID: 4UXL) resulted in obtaining 10 and 9 flavonoids, respectively, with higher docking scores than lorlatinib (Tables S1 and S2). From the docking analysis, the obtained flavonoids demonstrated favorable interactions with the ROS-1 catalytic binding pocket and did not exhibit Arg2032-mediated steric clashes with the MT ROS-1 kinase. The flavonoids were further escalated for molecular dynamics simulations to observe their behavior at physiological conditions and also to gain insight into the key residues required for selective ROS-1 inhibition. The representative structures for the 10 flavonoids were extracted from the stable MD trajectories (Figure 3) and the obtained flavonoids exhibited interactions with the residues of the WT and MT ROS-1 kinase as seen in previous studies [45,47]. Additionally, the flavonoids demonstrated no Arg2032-induced steric clashes and instead formed interactions with the residue via hydrophobic and van der Waals bonds (Figures S2 and S3).

Furthermore, the MD simulations were supplemented with MM/PBSA for computing the binding free energies of the respective complexes (Tables S3 and S4). Three (ST4119644, ST50837833 and ST096317) and two (ST4119644 and ST051039) flavonoids presented with better binding energies than lorlatinib with the WT and MT ROS-1 kinase, respectively (Tables S3 and S4). The flavonoid with *TimTec* ID: ST4119644 was observed as the common entity demonstrating better energy than lorlatinib with both the WT and MT ROS-1 kinase (Figure S4). Therefore, it was considered as a Hit compound. The binding interaction of our Hit in the ROS-1 catalytic pocket was compared with the interaction of lorlatinib. In the case of WT ROS-1, lorlatinib was observed to form hydrogen bonds with residues of the hinge region (Glu2027 and Met2029) and the solvent front (Gly2032) (Figure 5, Table 3), while Hit formed hydrogen bonding interactions with residues of the β-sheet (Lys1980) and DFG-motif (Gly2101) (Figure 5, Table 3). With MT ROS-1, lorlatinib retained hydrogen bonds with the aforementioned hinge residues and also with the mutated Arg2032 (Figure 6, Table 3), while Hit also retained its hydrogen bonding interaction with Lys1980 and shifted its orientation to form another hydrogen bond with residue Phe2103 of the DFG-motif (Figure 6, Table 3). Recent studies by Tian et al. reported about the catalytic pocket comprising key residues from the P-loop, β-sheet and the DFG-motif contributing to the high selectivity of compounds with ROS-1 over ALK [31,32]. In addition, Davare et al. also reported about the hydrogen bond with β-sheet residue Lys1980 in ROS-1/Cabozantinib interaction as acquired from MD simulations, conferring selectivity with ROS-1 [14].

**Figure 7:**
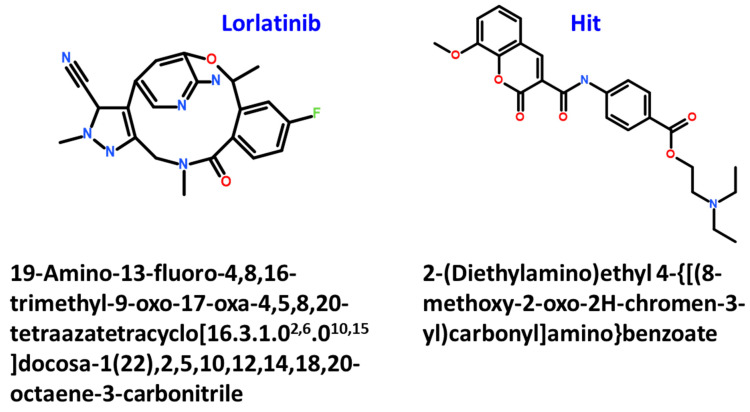
The 2D structures of lorlatinib and Hit flavonoid compound of ROS-1 tyrosine kinase.

The MM/PBSA calculations facilitate the decomposition of the BFE ΔG_bind_ into identifiable contributions. As observed from the BFE analysis, the van der Waals interactions provided the driving force for the flavonoids binding with WT and MT ROS-1 kinase systems (Tables S3 and S4). Moreover, the contribution of van der Waals interaction in the binding of Hit molecule with both WT and MT ROS-1 kinase was perceived as the highest than lorlatinib and other flavonoids. As described in a previous study, the van der Waals forces dominated the inhibitor binding and also determined the specificity with ROS-1 over ALK [32]. Therefore, from the above analysis, it can be interpreted that our Hit maybe selective for ROS-1 than ALK. Correspondingly, the identified flavonoids including the Hit molecule, also portray the pharmacophoric features required for ROS-1 inhibition, similar to that of lorlatinib (Figure 1 and Figure S5). Finally, the 2D chemical structures of lorlatinib and the Hit molecule were presented along with their IUPAC names (Figure 7). The in vitro profiling of our identified hit compounds can be performed by cell viability assays and their inhibitory IC_50_ values against different NSCLC cell lines (HCC78, H3122 and A549) [14] can be calculated. Although the in vitro studies of our identified flavonoid molecules are required to corroborate our findings, the structure-based pharmacophore modeling can be very valuable to design novel compounds as ROS-1 RTK inhibitors in the future. Additionally, our study epitomizes an essential platform for the identification of flavonoids as NSCLC therapeutics.

## 4. Materials and Methods

### 4.1. Receptor-Ligand Pharmacophore Model Generation

The structure-based pharmacophore model explores the catalytic site of the protein interaction with its bound co-crystallized ligand. The information obtained from this pharmacophore model helps in identifying key pharmacophoric features responsible for protein inhibition [48,49]. The structure of ROS-1 bound with its co-crystallized inhibitor, Lorlatinib (PDB ID: 4UXL, 2.40 Å) [16], was obtained from the Protein Data Bank (PDB) and considered for receptor-ligand pharmacophore generation. The Receptor-Ligand Pharmacophore Generation module implanted in Discovery Studio (DS) v.2018 was utilized to generate the model. Given a receptor-ligand interaction, this module works in two steps to create a selective pharmacophore model. In the first step, six pre-defined standard features, including hydrogen bond acceptor (HBA), hydrogen bond donor (HBD), positive ionizable (PI), negative ionizable (NI), ring aromatic (RA) and hydrophobic (Hy), are identified matching the interaction between the protein with its binding ligand [50,51]. The pharmacophore models are ranked in the second step and the model selectivity is estimated based on the genetic function approximation (GFA) model [51]. Subsequently, the model is chosen based on its selectivity score.

### 4.2. Validation of Receptor-Ligand Pharmacophore Model

The validation of the generated pharmacophore model is carried out to assess the pharmacophore’s ability to segregate active compounds from inactive ones [51]. The model generated was validated by the Güner-Henry (GH) approach [52], and the goodness of fit (GF) score was subsequently calculated in the range of 0 to 1, indicating the null and ideal model, respectively [46] according to the following formula:

$$\mathrm{GF}=\;\left(\frac{\mathrm{Ha}}{4\mathrm{HtA}}\right)\left(3A+\mathrm{Ht}\right)\;\times \;\left\{1-\frac{\mathrm{Ht}-\mathrm{Ha}}{D-A}\right\}$$

where D represents an external dataset of known active and inactive compounds of the protein while A denotes the active ROS-1 inhibitors, consequently, the Ht represents the hit molecules retrieved from D, and Ha signifies the active hit molecules obtained. The Ligand Pharmacophore Mapping tool complemented with the FAST algorithm embedded in DS was employed for computing the GF score.

### 4.3. Virtual Screening of TimTec Flavonoids Database

The 3D database of ligands was prepared by downloading and importing the Flavonoids library of molecules from the TimTec database (https://www.timtec.net/, accessed on 5 April 2021) consisting of 4560 compounds. The validated pharmacophore model was used as a 3D query for database screening to identify molecules as potential ROS-1 inhibitors satisfying the pharmacophoric features, utilizing the Ligand Pharmacophore Mapping module in DS. The potential compounds mapping the pharmacophoric features were further subjected to two filtering criteria- Lipinski’s rule of five (Ro5) [53] and Veber’s rule [54] by employing the Filter by Lipinski and Veber Rules tool in DS. The Ro5 suggests that the drug is absorbed well when the molecule has ≤5 HBD groups, ≤10 HBA groups, <500 Da molecular weight and a log P < 5. Veber’s rule entails the rotatable bonds of ≤10 for an orally bioavailable compound. Further filtering for pharmacokinetic properties was performed by absorption, distribution, metabolism, excretion and toxicity (ADMET) analysis for the obtained compounds utilizing the ADMET Descriptors module in DS. The ADMET tool is used to predict pharmacokinetic properties for compounds including human intestinal absorption, blood-brain barrier, aqueous solubility and hepatotoxicity. The drug-like compounds obtained from this criteria are escalated for molecular docking with the crystal structure of ROS-1 kinase.

### 4.4. Molecular Docking of Drug-Like Compounds with ROS-1 Tyrosine Kinase

Molecular docking of the drug-like compounds obtained from the above-mentioned filtering process was performed to generate their bioactive binding poses within the catalytic site of WT and MT ROS-1 kinase. The docking process was carried out by employing the genetic optimization for ligand docking (GOLD) v5.2.2 software [55], and hits were chosen based on clustering, Gold scores [56] and interactions with ROS-1. The 3D crystallographic structure retrieved from PDB, complexed with lorlatinib (PDB ID: 4UXL), was prepared with the Clean Protein module in DS. As the crystallographic structure of MT ROS-1 is not available, the Gly2032Arg mutation was modelled using the Build and Edit Protein tool implemented in DS. Prior to docking, the water molecules [57] and the bound ligand were removed, missing residues were supplemented and the protein was minimized. The performance of GOLD software was checked to ensure efficient docking of drug-like compounds by re-docking Lorlatinib into ROS-1 kinase. Subsequently, the Minimize Ligands protocol in DS was utilized to prepare ligands via minimization before docking with ROS-1. The grid co-ordinates were set as 42.32 (X), −19.55 (Y) and −4.86 (Z) with a radius of 7.00 Å for docking of drug-like compounds where lorlatinib was used as a reference. The potential flavonoids obtained from docking were subjected to molecular dynamics (MD) simulations to check their stability at the atomic level.

### 4.5. Molecular Dynamics Simulation and Binding Free Energy Calculations

MD simulation studies of the potential flavonoids obtained from the docking process were carried out for 50 ns to check their stability in water at the atomistic level in GROningen MAchine for Chemical Simulations (GROMACS) v2018 [58]. The docked complexes of the flavonoids with WT and MT ROS-1 kinase were used as initial coordinates for MD simulations by generating the protein and ligand topologies with CHARMm27 force field [59] and SwissParam [60], respectively. The system was first solvated with a dodecahedron water box using a TIP3P water model and subsequently neutralized by adding the Na^+^ counter ions. After the steepest descent energy minimization, a two-fold equilibration protocol was executed with NVT (constant number of particles, volume and temperature) and NPT (constant number of particles, pressure and temperature) of 500 ps each. Bond constraints and the geometry of water molecules during the simulation were monitored by the linear constraint solver (LINCS) [61] and SETTLE [62] algorithms, while the long-range electrostatic interactions were computed using the particle mesh Ewald (PME) [63]. The results obtained from MD studies were analyzed in DS as well as in visual molecular dynamics (VMD) [64] software package. The stability of the flavonoid molecules was checked by computing their RMSD values over the entire simulation production run, and their binding orientation in the ROS-1 pocket was analyzed. Additionally, the interaction of the hits with WT and MT ROS-1 catalytic residues was examined and compared with the selective inhibitor, lorlatinib. The presence or absence of steric clashes with the Arg2032 residue of the MT ROS-1 kinase was examined and analyzed. The g_mmpbsa tool of GROMACS was utilized to compute the binding free energy scores of the MD-derived complexes [65]. A total of 50 frames were selected evenly from the last 10 ns (40–50 ns) of stable MD trajectories and the free energy of binding ∆G_bind_ was calculated according to the following equation of molecular mechanics Poisson–Boltzmann surface area (MM/PBSA):

$$\Delta G_{\mathrm{bind}}={\;G}_{\mathrm{complex}}-\left(G_{\mathrm{protein}}+{\;G}_{\mathrm{ligand}}\right)$$

## 5. Conclusions

ROS-1 has emerged as a therapeutic target in various malignancies including NSCLC. In the present study, a structure-based pharmacophore model exploiting the crystal structure of ROS-1 with its bound selective inhibitor, lorlatinib, was generated and used for virtual screening of the TimTec flavonoids database. The obtained flavonoids were filtered for their drug-likeness, and a total of 19 flavonoids were subjected to molecular docking both with the WT and MT ROS-1 kinase domain. The flavonoids displayed interactions with key residues of the binding pocket. In addition, no steric clashes were observed with the Arg2032 residue of the MT ROS-1 kinase domain. Subsequently, molecular dynamics simulations combined with binding free energy calculations of flavonoids, with both the WT and MT systems, revealed one flavonoid (Hit) depicting higher energy than lorlatinib. The Hit compound was observed to inhibit ROS-1 by extending into the DFG-motif selectivity pocket than lorlatinib’s binding with the hinge region residues. Our results depicted that the interaction of the Hit compound with DFG-motif was responsible for contributing significantly to the binding affinity with ROS-1. Additionally, the identification of flavonoids signifies a quintessential platform for future drug discovery studies against ROS-1 fusion-positive NSCLC tumors. We, therefore, believe that the scaffolds of our identified flavonoids can act as frameworks for selective drug optimization against ROS-1 kinase.

## Figures and Tables

**Table 1:** Structure-based pharmacophore model summary with its generated features.

Pharmacophore Models	Number of Features	Feature Set *	Selectivity Score
Pharmacophore_01	4	ADHH	7.0747

* A: hydrogen bond acceptor (HBA); D: hydrogen bond donor (HBD); H: hydrophobic (Hy).

**Table 2:** Decoy set validation of Pharmacophore_01 from an external database composed of active ROS-1 inhibitors and decoy set molecules.

Set No.	Parameters	Values
1	Total number of compounds in the database (D)	78
2	Total number of active compounds in the database (A)	20
3	Total number of hits retrieved by pharmacophore model from the database (Ht)	25
4	Total number of active compounds in the hit list (Ha)	20
5	% Yield of active ((Ha/Ht) × 100)	80
6	% Ratio of actives ((Ha/A) × 100)	100
7	False negatives (A-Ha)	0
8	False positives (Ht-Ha)	5
9	Goodness of fit score (GF)	0.77

**Table 3:** Molecular interactions between the compounds (lorlatinib and Hit) and the active site residues of wild-type (WT) and mutated (MT) ROS-1 kinase acquired from stable molecular dynamics (MD) trajectories.

Complex	Hydrogen Bond Interactions (Distance in Å)	Carbon Hydrogen Bond Interactions	Hydrophobic (π) Interactions	Van der Waals Interactions
Lorlatinib (with WT ROS-1)	Glu2027 (1.96), Met2029 (2.21), **Gly2032** (3.05)	Leu1951, Met2029, Leu2030, Gly2101	Val1959, Ala1978, Lys1980, Leu2026, Leu2028	Gly1952, Leu2010, Leu2028, Asp2033, Arg2083, Asp2102,
Hit (with WT ROS-1)	Lys1980 (2.81), Gly2101 (2.03)	Met2029	Leu1951, Met2001, Leu2010, Leu2028, Leu2086, Phe2103	Ser1953, Glu1961, Glu1997, Leu2000, Phe2004, Ile2009, Leu2026, Glu2030, **Gly2032**, Asp2102
Lorlatinib (with MT ROS-1)	Glu2027 (1.81), Met2029 (1.95), **Arg2032** (2.12)	Leu1951, Met2029	Val1959, Ala1978, Lys1980, Leu2026, Leu2086	Gly1952, Leu2010, Leu2028, Glu2030, Gly2031, Asp2033, Asn2084, Asp2102
Hit (with MT ROS-1)	Lys1980 (2.97), Phe2103 (1.85)	Ala2106	Leu1951, Glu1997, Met2001, Phe2004, Leu2026, Leu2028, **Arg2032**, Phe2103	Gly1952, Val1959, Ala1978, Leu2000, Leu2010, Met2029, Phe2075, Leu2086, Gly2101, Asp2102, Gly2104

## Data Availability

Data are contained within the article.

## Supplementary Materials

The following are available online at, Figure S1. Overlay of the docked pose (green) of lorlatinib with its crystal structure conformation (pink) in PDB ID: 4UXL. Figure S2. Binding interaction of flavonoids with key residues of the wild-type (WT) ROS-1 kinase domain. The flavonoids are represented as sticks. Hydrogen bonds are indicated as green dashed lines, hydrophobic interactions are shown as pink and purple spheres and the van der Waals interactions are displayed as light green spheres. Figure S3. Binding interaction of flavonoids with key residues of the mutated (MT) ROS-1 kinase domain. The flavonoids are represented as sticks. Hydrogen bonds are indicated as green dashed lines, hydrophobic interactions are shown as pink, purple and orange spheres and the van der Waals interactions are displayed as light green spheres. Figure S4. Binding free energy (BFE) analysis of (A) wild-type (WT) and (B) mutated (MT) ROS-1 systems. Figure S5. Alignment of the identified flavonoids with the pharmacophoric features. All flavonoids ((B)–(J)) including the (A) Hit compound represent the HBA (hydrogen bond acceptor), Hy (hydrophobic) and HBD (hydrogen bond donor) features of *Pharmacophore_01*. Table S1. The docking scores and intermolecular interactions of lorlatinib and flavonoids from *TimTec* database with ROS-1 wild-type (WT) tyrosine kinase domain. Table S2. The docking scores and intermolecular interactions of lorlatinib and flavonoids from *TimTec* database with ROS-1 mutated (MT) tyrosine kinase domain. Table S3. Binding free energy scores of the identified flavonoids with wild-type (WT) ROS-1 kinase computed through MM/PBSA methodology. Table S4. Binding free energy scores of the identified flavonoids with mutated (MT) ROS-1 kinase computed through MM/PBSA methodology.

## Abbreviations

ADMET	absorption: distribution, metabolism, excretion, toxicity
AKT	protein kinase B
ALK	anaplastic lymphoma kinase
BFE	binding free energy
DFG	Asp-Phe-Gly
EGF	epidermal growth factor
FDA	food and drug administration
GF	goodness of fit
GFA	genetic function approximation
GH	Güner-Henry
GOLD	genetic optimization for ligand docking
GROMACS	GROningen MAchine for Chemical Simulations
HBD	hydrogen bond donor
HBA	hydrogen bond acceptor
Hy	hydrophobic
IC_50_	half maximal inhibitory concentration
LINCS	LINear Constraint Solver
LUAD	lung adenocarcinoma
LUSC	lung squamous cell carcinoma
MD	molecular dynamics
MT	mutant
mTOR	mammalian target of rapamycin
MM/PBSA	molecular mechanics/poisson-boltzmann surface area
NI	negative ionizable
Nrf2	nuclear factor erythroid 2-related factor 2
NVT	constant number of particles, volume and temperature
NPT	constant number of particles, pressure and temperature
NSCLC	non-small cell lung cancer
PDB	protein data bank
PI	positive ionizable
PI3K	phosphoinositide 3-kinase
P-loop	phosphate binding loop
PME	Particle mesh Ewald
RA	ring aromatic
RTK	receptor tyrosine kinase
ROS-1	c-ros oncogene 1
Ro5	rule of five
RMSD	root mean square deviation
VMD	visual molecular dynamics
WT	wild-type
