# Supplementary material for: Identification of Flavonoids as Putative ROS-1 Kinase Inhibitors Using Pharmacophore Modeling for NSCLC Therapeutics
Source: Molecules. 2021 Apr 7;26(8):2114. doi: 10.3390/molecules26082114 (PMC8067712; doi:10.3390/molecules26082114)
Supplement: Supplementary file 1 [file molecules-26-02114-s001.pdf]

molecules

Supplementary Material:

Identification of Flavonoids as Putative ROS-1 Kinase Inhibitors using Pharmacophore Modeling for NSCLC therapeutics

Shraddha Parate[1], Vikas Kumar[2], Jong Chan Hong[1,*] and Keun Woo Lee[2,*]

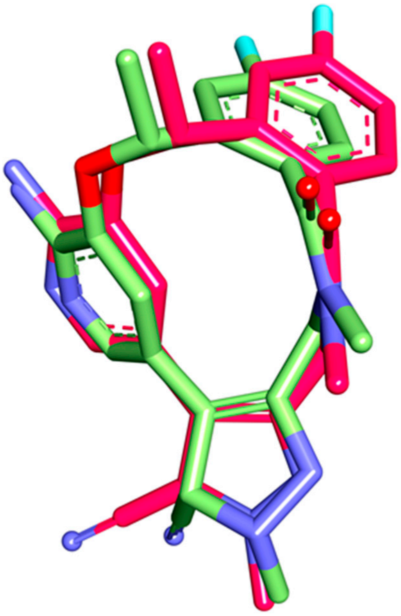

Figure S1. Overlay of the docked pose (green) of lorlatinib with its crystal structure conformation (pink) in PDB ID: 4UXL.

Table S1. The docking scores and intermolecular interactions of lorlatinib and flavonoids from *TimTec* database with ROS-1 wild-type (WT) tyrosine kinase domain.

TimTec ID No.	Gold Score	Hydrogen Bond Interactions	Hydrophobic and van der Waals Interactions
ST4119644	72.80	Lys1980, Met2029	Leu1951, Val1959, Glu1961, Ala1978, Lys1980, Glu1997, Met2001, Leu2010, Leu2026, Leu2028, Glu2030, **Gly2032**, Leu2086, Gly2101, Asp2102, Gly2104
ST051039	61.78	Lys1980, Glu1997, Met2029	Leu1951, Val1959, Ala1978, Met2001, Leu2010, Ile2024, Leu2026, Glu2027, **Gly2032**, Leu2086, Gly2101
ST052370	52.40	Met2029, Asp2033	Leu1951, Val1959, Ala1978, Lys1980, Met2001, Leu2010, Leu2026, Glu2027, Leu2028, Glu2030, **Gly2032**, Leu2086, Gly2101, Asp2102
ST077081	51.68	Lys1980, Gly2101	Leu1951, Val1959, Ala1978, Leu2010, Leu2026, Leu2028, Met2029, Glu2030, **Gly2032**, Leu2086, Asp2102
ST063011	51.05	Lys1980, Met2029	Leu1951, Val1959, Ala1978, Glu1997, Met2001, Leu2010, Leu2026, Glu2027, Leu2028, Glu2030, **Gly2032**, Leu2086, Gly2101, Asp2102, Phe2103, Gly2104
ST4146854	50.31	Lys1980, Met2029	Leu1951, Val1959, Ala1978, Glu1997, Leu2010, Leu2026, Glu2027, Glu2030, **Gly2032**, Leu2086, Gly2101
ST50843253	49.01	Met2029	Leu1951, Gly1952, Ser1953, Gly1954, Val1959, Ala1978, Lys1980, Leu2010, Leu2026, Glu2027, Glu2030, Leu2028, **Gly2032**, Leu2086
ST50837833	48.11	Lys1980, Met2029	Leu1951, Val1959, Glu1961, Ala1978, Leu2010, Leu2026, Glu2027, Glu2030, **Gly2032**, Leu2086, Gly2101, Asp2102
ST50707205	47.07	Lys1980	Leu1951, Ser1953, Gly1954, Val1959, Ala1978, Met2001, Leu2010, Leu2026, Leu2028, Met2029, **Gly2032**, Arg2083, Asn2084, Leu2086, Gly2101, Asp2102, Phe2103
ST096317	46.15	Lys1980, Glu2027, Met2029	Leu1951, Gly1952, Gly1954, Val1959, Ala1978, Leu2010, Leu2026, Leu2028, Glu2030, **Gly2032**, Leu2086
Lorlatinib	43.74	Glu2027, Met2029, **Gly2032**	Leu1951, Gly1952, Gly1954, Val1959, Ala1978, Lys1980, Leu2010, Leu2026, Leu2028, Asp2033, Leu2086

Table S2. The docking scores and intermolecular interactions of lorlatinib and flavonoids from *TimTec* database with ROS-1 mutated (MT) tyrosine kinase domain.

***TimTec* ID No.**	**Gold Score**	**Hydrogen Bond Interactions**	**Hydrophobic and van der Waals Interactions**
ST4119644	73.62	Lys1980, Met2029	Leu1951, Gly1954, Val1959, Glu1961, Ala1978, Lys1980, Glu1997, Met2001, Leu2010, Leu2026, Glu2027, Leu2028, Glu2030, **Arg2032**, Leu2086, Gly2101, Asp2102, Phe2103, Gly2104
ST051039	64.34	Met2029, Gly2101	Leu1951, Val1959, Ala1978, Lys1980, Leu2010, Leu2026, Leu2028, Glu2030, **Arg2032**, Leu2086
ST50843253	56.37	Met2029, **Arg2032**	Leu1951, Gly1952, Val1959, Ala1978, Leu2026, Glu2027, Leu2028, Met2029, Leu2010, Glu2030, **Arg2032**, Asp2033, Arg2083, Leu2086
ST052370	55.49	Met2029	Leu1951, Gly1952, Val1959, Ala1978, Leu2026, Leu2028, Glu2030, **Arg2032**, Asp2033, Arg2083, Asn2084, Cys2085, Leu2086, Asp2102
ST077081	53.62	Lys1980, Gly2101	Leu1951, Val1959, Ala1978, Leu2010, Leu2026, Leu2028, Met2029, **Arg2032**, Leu2086, Asp2102
ST50837833	51.49	Lys1980	Leu1951, Val1959, Ala1978, Leu2026, Leu2028, Met2029, Glu2030, **Arg2032**, Leu2086
ST50707205	51.45	Lys1980	Leu1951, Gly1952, Gly1954, Ser1953, Val1959, Ala1978, Leu2010, Leu2026, Leu2028, Met2029, Glu2030, **Arg2032**, Asp2033, Arg2083, Asn2084, Leu2086, Asp2102, Phe2103
ST4146854	50.24	Met2029, **Arg2032**	Leu1951, Gly1952, Val1959, Ala1978, Leu2028, Glu2030, Gly2031, **Arg2032**, Asp2033, Arg2083, Leu2086
ST063011	50.1	Lys1980, Met2029	Leu1951, Gly1954, Ala1955, Val1959, Ala1978, Leu2010, Leu2026, Glu2027, Leu2028, Glu2030, **Arg2032**, Leu2086, Gly2101, Asp2102
Lorlatinib	49.37	Glu2027, Met2029, **Arg2032**	Leu1951, Gly1952, Gly1954, Val1959, Ala1978, Lys1980, Leu2010, Leu2026, Leu2028, Asp2033, Leu2086, Asp2102
ST096317	47.15	Lys1980, Glu2027, Met2029	Leu1951, Gly1952, Gly1954, Val1959, Ala1978, Lys1980, Leu2010, Leu2026, Leu2028, **Arg2032**, Leu2086

Table S3. Binding free energy scores of the identified flavonoids with wild-type (WT) ROS-1 kinase computed through MM/PBSA methodology.

TimTec ID	van der Waals (kJ/mol)	Electrostatic (kJ/mol)	Polar solvation (kJ/mol)	SASA energy (kJ/mol)	Binding energy ΔG_{bind} (kJ/mol)
ST4119644	-206.983+/-17.338	-74.699+/-18.331	214.499+/-24.603	-24.502+/-1.074	-91.685+/-20.795
ST50837833	-156.498+/-9.550	-2.332+/-4.885	87.358+/-12.881	-17.287+/-0.860	-88.759+/-16.448
ST096317	-124.560+/-8.289	-22.887+/-5.553	72.266+/-8.211	-13.562+/-0.770	-88.744+/-9.773
Lorlatinib	-182.556+/-10.399	-30.294+/-6.600	142.724+/-12.717	-18.117+/-0.778	-88.244+/-12.933
ST50707205	-175.441+/-17.394	-30.543+/-10.584	140.517+/-20.621	-19.406+/-1.240	-84.873+/-14.681
ST052370	-158.976+/-13.663	-25.749+/-18.882	126.783+/-36.610	-18.315+/-1.358	-76.258+/-12.242
ST4146854	-152.033+/-12.001	-35.504+/-8.556	133.149+/-30.010	-17.238+/-1.140	-71.625+/-20.226
ST063011	-140.414+/-14.603	-16.669+/-15.958	103.442+/-25.036	-16.511+/-1.377	-70.151+/-10.935
ST077081	-126.006+/-11.536	-34.127+/-14.307	117.876+/-16.187	-15.954+/-0.952	-58.211+/-11.421
ST051039	-158.842+/-9.316	-71.393+/-27.799	191.585+/-31.463	-19.234+/-0.794	-57.884+/-15.203
ST50843253	-112.840+/-11.108	-25.897+/-13.760	105.361+/-19.246	-14.422+/-0.757	-47.798+/-16.563

Table S4. Binding free energy scores of the identified flavonoids with mutated (MT) ROS-1 kinase computed through MM/PBSA methodology.

TimTec ID	van der Waals (kJ/mol)	Electrostatic (kJ/mol)	Polar solvation (kJ/mol)	SASA energy (kJ/mol)	Binding energy ΔG_{bind} (kJ/mol)
ST4119644	-225.602+/-12.106	-14.952+/-13.683	163.374+/-20.851	-25.854+/-1.088	-103.035+/-16.223
ST051039	-163.533+/-11.452	-100.917+/-8.289	204.277+/-12.685	-20.357+/-1.228	-80.531+/-12.060
Lorlatinib	-171.545+/-11.137	-26.433+/-9.173	140.720+/-16.636	-18.246+/-1.187	-75.505+/-12.053
ST096317	-141.478+/-8.771	-9.587+/-8.261	93.500+/-14.323	-15.599+/-0.751	-73.165+/-11.518
ST077081	-150.416+/-10.061	-50.242+/-14.394	148.162+/-14.466	-16.474+/-0.731	-68.970+/-12.966
ST50837833	-158.519+/-8.741	-5.606+/-6.718	112.490+/-16.206	-17.327+/-0.759	-68.962+/-10.945
ST052370	-164.272+/-10.392	4.860+/-6.486	110.367+/-11.953	-19.445+/-0.748	-68.490+/-12.688
ST50707205	-192.144+/-11.731	-38.256+/-11.542	187.196+/-22.847	-20.629+/-1.010	-63.832+/-17.966
ST063011	-150.507+/-7.692	-23.358+/-7.686	128.758+/-14.294	-17.195+/-0.791	-62.302+/-11.448
ST4146854	-131.148+/-9.103	-29.211+/-9.581	127.515+/-14.325	-15.781+/-1.071	-48.626+/-12.066
ST50843253	-143.703+/-17.785	-1.242+/-10.677	115.513+/-41.590	-16.707+/-1.560	-46.139+/-22.786

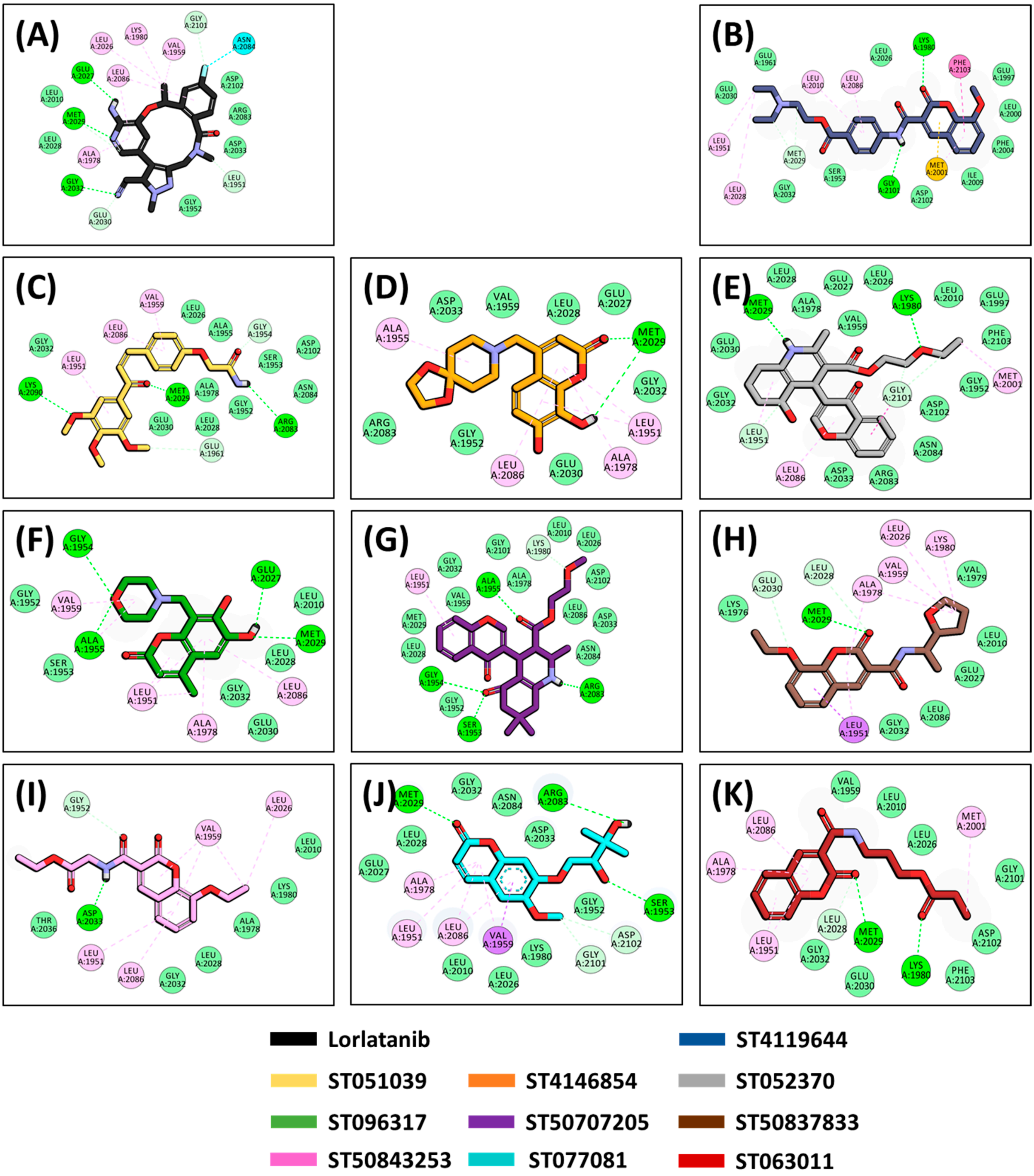

Figure S2. Binding interaction of flavonoids with key residues of the wild-type (WT) ROS-1 kinase domain. The flavonoids are represented as sticks. Hydrogen bonds are indicated as green dashed lines, hydrophobic interactions are shown as pink and purple spheres and the van der Waals interactions are displayed as light green spheres.

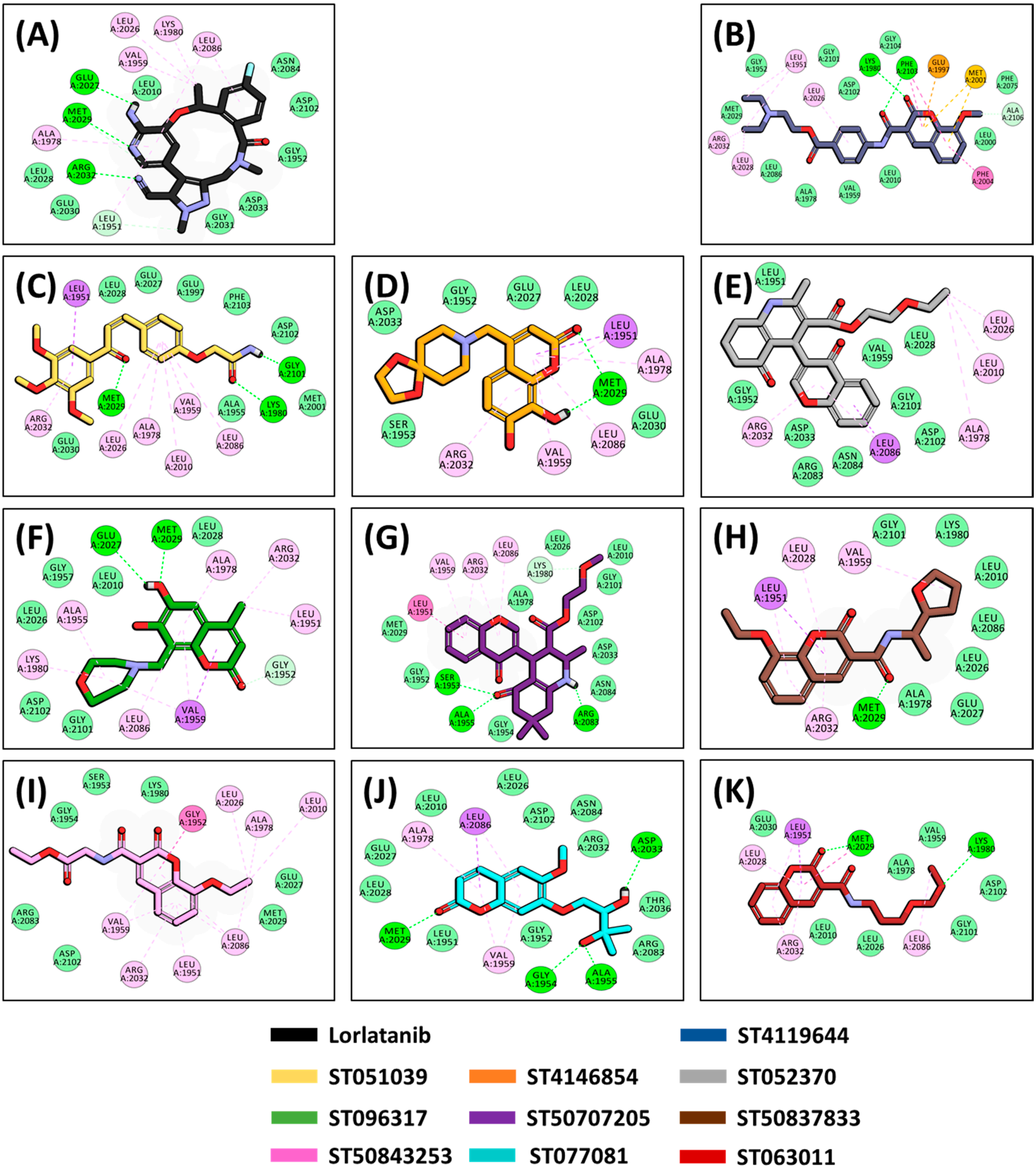

Figure S3. Binding interaction of flavonoids with key residues of the mutated (MT) ROS-1 kinase domain. The flavonoids are represented as sticks. Hydrogen bonds are indicated as green dashed lines, hydrophobic interactions are shown as pink, purple and orange spheres and the van der Waals interactions are displayed as light green spheres.

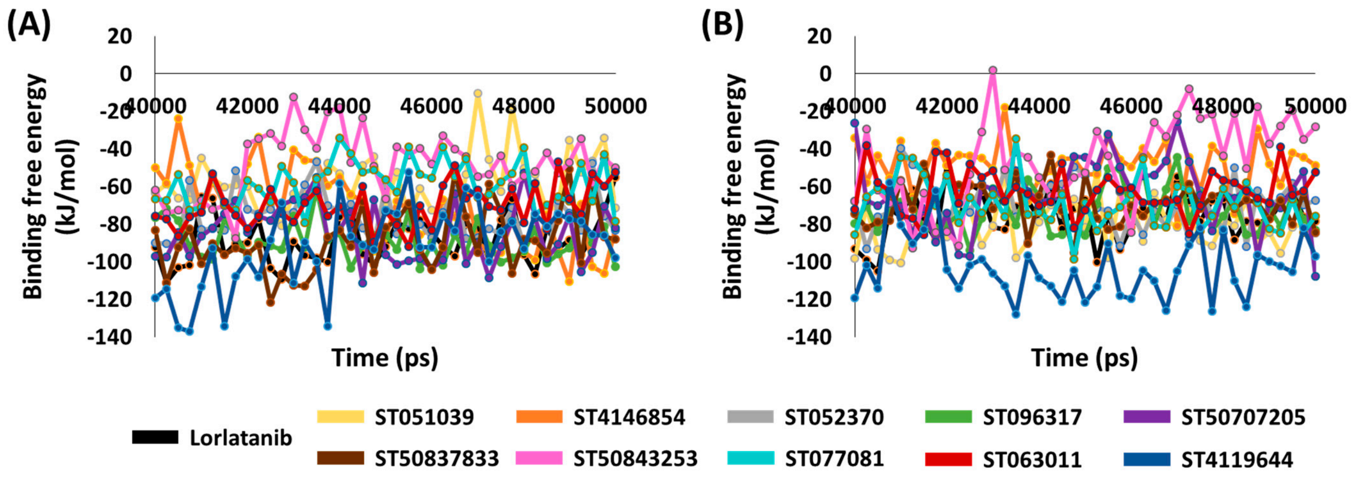

Figure S4. Binding free energy (BFE) analysis of (A) wild-type (WT) and (B) mutated (MT) ROS-1 systems.

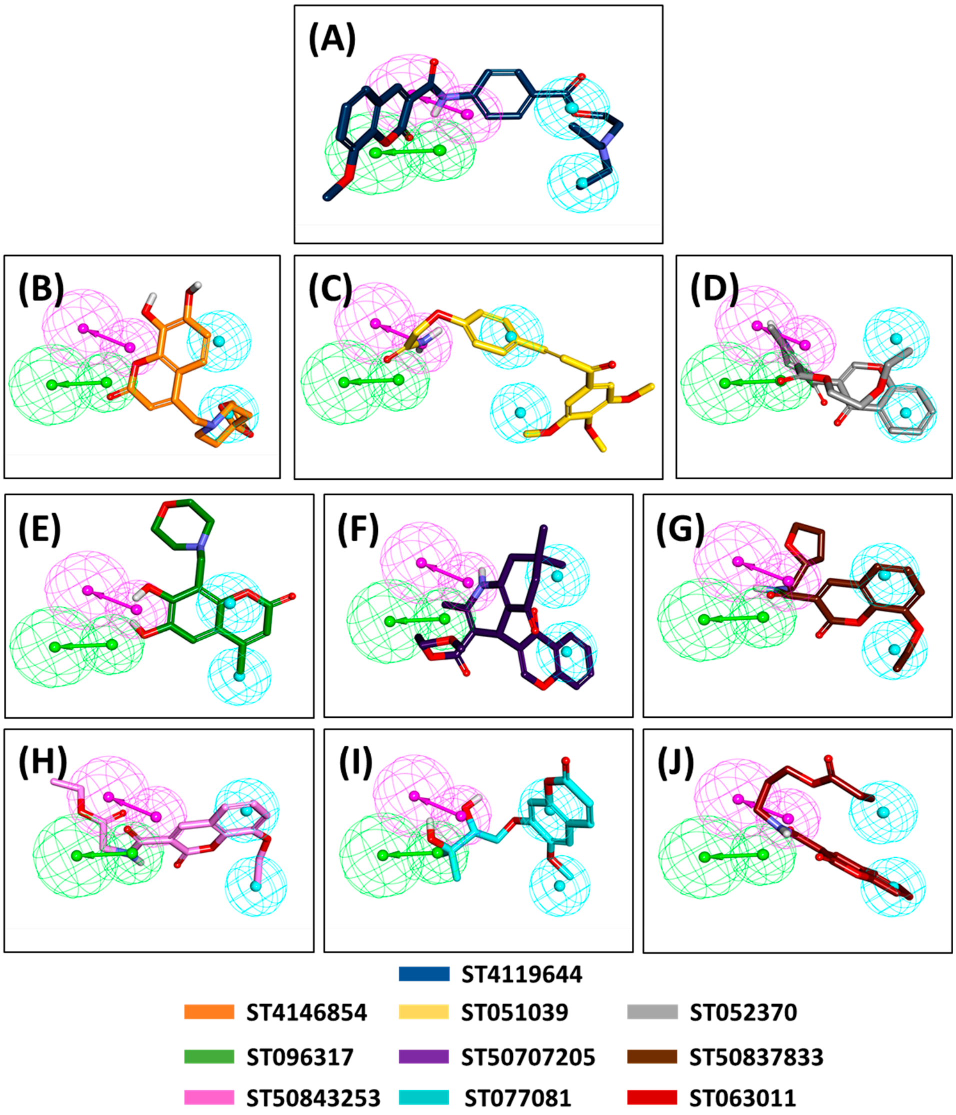

Figure S5. Alignment of the identified flavonoids with the pharmacophoric features. All flavonoids ((B) - (J)) including the (A) Hit compound represent the HBA (hydrogen bond acceptor), Hy (hydrophobic) and HBD (hydrogen bond donor) features of *Pharmacophore_01*.
